# Why do few Medicare beneficiaries switch their Part D prescription drug plans? Insights from behavioral sciences

**DOI:** 10.1057/s41271-025-00618-1

**Published:** 2025-12-23

**Authors:** Nathan Hodson, Wändi Bruine de Bruin

**Affiliations:** University of Southern California, Los Angeles, USA

**Keywords:** Medicare, Medicare Part D, Behavioral economics, Health economics, Sludge, Prescription drug plan, United States

## Abstract

Medicare Part D innovatively included a market in public health insurance in the United States. Proponents argued that increased competition would drive better value for beneficiaries, but others feared that beneficiaries would struggle to navigate the complex program. Understanding how Part D beneficiaries choose between plans allows us to evaluate the extent to which Medicare Part D succeeds at increasing value to beneficiaries and where there is scope to support beneficiaries. Many Part D beneficiaries are sensitive to price cues in relation to pharmacy choice and medication adherence, yet frequently overpay for their plans. Empirical literature suggests that behavioral aspects including information overload, low salience, low trust, and practical ‘sludge’ all partly contribute to failure to switch. We propose solutions to address these barriers based on behavioral insights.

## Key messages

- Part D plan choices are influenced by information overload, low salience of the benefits of switching, administrative burdens or ‘sludge’, as well as low confidence and trust.
- Possible policy responses could include setting defaults, reducing the number of options, reducing plan selection to every 5 years, and offering trusted advisors.
- This viewpoint demonstrates how insights from behavioral science can be applied to understand health policies and these principles can be applied more widely.

## Introduction

The Medicare Modernization Act 2003 was the largest expansion of public health insurance in the United States since the 1960s. Medicare Part D offered Americans with disabilities and those aged 65 and over subsidized prescription drug insurance [1]. The Act gave private insurance providers a prominent role in delivering prescription drug insurance coverage under Medicare Part D [2]. In 2022, 49 million Americans had Part D plans [3].

Beneficiaries have the option to switch their Medicare Part D plan during an annual enrolment period, when their plan’s features may change. They have the option to keep the plan they already had or they can choose to switch to another plan, with a large number of alternative plans being available [4]. Plan features vary, offering different premiums, deductibles, co-pays for specific drugs, and preferred pharmacies [4]. As patients’ year-to-date spending on prescription drugs increases, the amount covered by insurers varies [5]. Patients are generally responsible for ‘the deductible’ or the first few hundred dollars. After paying the deductible there is an initial coverage phase where beneficiaries’ contributions are lower. Subsequently, patients’ contributions increase again during a ‘coverage gap’ or ‘donut hole’ phase. Finally, in the ‘catastrophic coverage’ phase the federal government covers most of the costs, the insurer provides some, and patients pay relatively little. Over the last 10 years the coverage gap has been phased out and it was completely closed from January 2025 [6].

Proponents of the Part D individual marketplace argued that giving people many plans to choose from would allow them to select the best plan for their situation, including the drugs they take and the pharmacies they prefer, while also encouraging competition between insurance companies [7, 8]. Yet many people fail to change plan when doing so would save them money. In this viewpoint, we explore the reasons for non-switching drawing on insights from behavioral economics.

### How often do beneficiaries switch their Medicare Part D plans?

Only about 10% of beneficiaries switch plans each year [9, 10]. Among Part D beneficiaries eligible for the Low Income Supplement only 16% actively choose their plan, the rest stick with the plan they were initially offered [11]. At any given time, two thirds of beneficiaries eligible for the Low Income Supplement remain in the plan they were initially defaulted into [11].

Not switching can result in beneficiaries overpaying because over time their plans change and gradually provide less benefits and higher costs [12]. This means that beneficiaries end up with plans that are not best for reducing the costs of their prescription drugs and this can have significant financial implications [13]. In 2009, only 5.2% of beneficiaries had financially optimal plans, overspending on average $368 per year, and some overspending much more [14]. Moreover, one in three low-income beneficiaries is underinsured, resulting in higher out-of-pocket costs [12]. Failure to switch out of an expensive plan can also have health implications because mismatches between coverage and prescriptions are associated with medication non-adherence [15].

### Why do few beneficiaries switch plans?

Although infrequent switching may seem to suggest that beneficiaries are insensitive to financial incentives, evidence suggests that this is not the case [16–18]. First, an overwhelming proportion of eligible people chose to enroll in Medicare Part D when it was first introduced, benefiting their household finances [16]. Non-enrollment was largely accounted for by risk preferences in that people who tended to like taking risks were underrepresented among early enrollees [17]. Second, unsubsidized beneficiaries tend to switch to pharmacies that are covered by their plans, responding to financial penalties averaging $146 annually [19]. Third, beneficiaries’ medication adherence increased after enrolling in Part D [18, 20]. For example, a study of 114,766 seniors without drug benefits found that after enrolling in Part D, adherence to common preventive health medications such as statins and simple anticoagulants increased [18]. Fourth, when beneficiaries’ annual costs reach the coverage gap, their medication adherence falls by around 6% [18], even for insulin and cancer-related prescriptions [15, 20]. Among Part D beneficiaries with additional insurance for the coverage gap, reaching the coverage gap was associated with an adherence reduction of only 3%, compared with a 14% reduction in adherence among those without additional coverage [21]. Together these findings suggest that beneficiaries respond to changes in how much they have to pay for their prescription drugs and so it may seem surprising that relatively few switch plans each year.

We explored why many Medicare Part D beneficiaries overpay for Part D plans which are ill-suited to their needs and suggest potential barriers to plan selection. We interpreted the findings through the lens of behavioral science, which combines economics and psychology. Over the last 50 years behavioral scientists have shown that people may sometimes make suboptimal decisions, due to having limited time, motivation, or ability [22, 23]. Behavioral science has suggested policies for addressing these issues and helping people with their decisions [24].

### Does information overload prevent beneficiaries from switching plans?

It is a common finding in behavioral science that people say they want a lot of options, yet many options leaves them too overwhelmed to actually make a choice [25]. The 2006 Retirement Perspective Survey found that 77% of respondents agreed that it was useful that there were more companies to choose from, while 71% also endorsed the idea that there were too many choices [26]. Indeed, Part D is commonly perceived as having too many options and this excess may lead to choice avoidance [26–28].

Studies of hypothetical Part D choices suggest that reducing the number of options in the Medicare Plan Finder can potentially help. The Plan Finder is a web-based choice tool that asks beneficiaries to enter their preferred pharmacies and the drugs that they take [29]. Three randomized experiments asked American adults to make hypothetical choices and found that they were more likely to choose cheaper plans when shown a version of the Medicare Plan Finder that simplified financial information by providing one personalized annual cost estimate instead of separate premiums, deductible, co-pays [30].

While simplifying the Medicare Plan Finder could help some beneficiaries who use it to make better choices, many beneficiaries do not engage with the Part D Finder [28]. Thus, it still remains to be seen whether simplifying the Part D Finder will change beneficiaries’ willingness to use it, and improve their likelihood of switching to financially optimal plans.

### Does paperwork sludge prevent beneficiaries from switching plans?

Another insight from behavioral science is that excessive paperwork costs—also called ‘sludge’—prevent people from undertaking behaviors that are good for them [31–33]. Sludge could stop people making the choice to switch away from financially suboptimal plans. Therefore, attempts have been made to provide beneficiaries with support in navigating the complexity of comparing and switching plans.

In one study running from 2008 to 2010, trained pharmacy students delivered 1300 one-on-one Part D plan consultations. Of the beneficiaries who agreed to free consultations, 29% were already enrolled in the lowest cost plan, 39% were overpaying and accepted help to switch to a more affordable plan, but 33% were overpaying and declined help to switch to a more affordable plan [34]. Additionally, a randomized-controlled trial evaluated an internet-based choice tool offering personalized Part D plan suggestions with or without additional expert advice, in comparison to a control group that received no intervention. Over 29,000 people were invited but only 1185 enrolled in the study, perhaps suggesting a reluctance to participate use this type of tool. Among the participants who received the choice tool with expert advice 37% switched plans, compared to 29% of those receiving choice tool without expert advice, and 28% of those in the control group [35]. Thus, in each group, most participants did not switch. This study does not report clearly whether those who did not switch already had the best plan or not, but it is unlikely given the large proportions who tend to overpay [14, 36]. Similar reluctance to switch to more affordable plans was found in other studies. For example, when student pharmacists provided guidance to 123 Part D beneficiaries, 90% stood to save money, for a median amount of $98, but only 45% took up the offer to switch [37].

Thus, providing beneficiaries with support has shown some success in helping them navigate choices to switch. The uptake of both support to review one’s options and support to switch to a cheaper option, however, is low.

### Does low salience of the benefits of switching prevent beneficiaries from switching plans?

The benefits of switching may not catch beneficiaries’ attention, in keeping with the insight from behavioral science that ‘salience’ is important [38]. In one randomized study of beneficiaries, an intervention group received a letter with personalized cost-saving information, which increasing switching compared with a control group that received no letter [31]. Yet, only 28% who received the letter switched compared with 17% in the control group [31]. Another study found that beneficiaries were more likely to switch if last year’s overspending was made salient to them [39]. These findings are in keeping with the finding that beneficiaries who faced a large increase of at least $120 per year had higher rates of switching, though most still did not switch [9].

Together these studies suggest that making the benefits of switching salient somewhat increases the number of beneficiaries who switch plans. However, even when the benefits of switching are made salient, most Part D beneficiaries still do not switch.

### Do lack of confidence and trust prevent beneficiaries from switching plans?

Behavioral science has long indicated that people need self-efficacy, or perceived confidence in their ability, to engage with difficult situations [40]. Even people with high numerical abilities may obtain poor financial outcomes if they have low confidence in their numerical abilities—perhaps because they fail to engage with their financial decisions [41]. Moreover, many older people lack the confidence that they can make decisions that involve numbers [42]. Interviews with Medicare Part D beneficiaries have indeed reported low confidence in their own ability to make plan choices [43]. People who have low confidence in their abilities often prefer to make choices with support from sources they trust [44]. Interviews with Medicare Part D recipients have found that not trusting anyone can undermine switching [7]. However, many Part D beneficiaries have reported trusting their pharmacists’ advice saying, for example, “I trust him overall because he does a really good job. He gets it…” [8]. Trust in advisors also influenced their relationships with insurers, “First of all, I knew him, so I already trusted him … and so the trust factor was real huge. I’d heard of the company that he was talking about, Physicians Mutual, that was a big thing” [8].

Interviews with beneficiaries also found that some trusted specific insurance companies, saying they had been “looking for an insurance company that I thought would work, knowing that I wanted to stick with (…), if at all possible to support the local, we’ve dealt with them all these years, didn’t really want to switch to a big name pharmacy” [8]. In focus groups, beneficiaries also talked about trusting specific insurance companies: “I have a faith in, I like, Blue Cross Blue Shield. And I do not like United Health” [43]. These interview and focus group studies reveal a factor which quantitative models and hypothetical experiments appear to have potentially overlooked: some Part D beneficiaries who lack confidence in their own abilities may distrust advice, unless it comes from sources they know.

### What insights does behavioral science have for policy makers who want to promote Part D switching?

Studies of Medicare Part D beneficiaries suggest that Part D plan choices are influenced by information overload, low salience of the benefits of switching, administrative burdens or ‘sludge’, as well as low confidence and trust. There may be other factors that have not yet been investigated. For example, the behavioral science literature suggests that people like sticking with their default because it is easy and allows them to not make a choice at all [45]. There is also evidence that people may worry about any switch as risking a relative ‘loss’ [46]. Thus, in-depth interviews and follow-up surveys are needed to gain a complete picture of part D plan beneficiary decision-making [47]. Such evidence could provide insights about barriers to plan choices, which policymakers may want to address [24]. Based on the evidence reviewed thus far, we will discuss three possible policy responses: setting defaults, reducing the number of options, reducing plan selection to every five years, and offering trusted advisors.

First, reducing the number of plans beneficiaries can initially choose would be another strategy for promoting better choices. It overcomes the problem of having to select one default. While people of all ages often say they want choice, older adults tend to prefer fewer options than younger adults [48]. Moreover, older adults perform better when being presented with a smaller choice set [49]. A ‘tournament’ strategy that shows smaller subsets of options in multiple rounds rather than all options at once may improve older adults’ decisions [50]. Nevertheless, some beneficiaries may still be hesitant to make choices, due to low confidence in their abilities.

Second, providing access to trusted advisors could be another strategy for promoting better choices. Older adults are more likely than younger adults to turn to financial advisors [51]. Older adults prefer to delegate some complex decisions to others to reduce their information overload [52–54]. Such preferences may also reflect older adults’ lower confidence in their abilities [55]. However, advisors may need to be paid, and some beneficiaries may not be able to come up with the money.

Third, another way to reduce the burden on beneficiaries is to offer plan choices less often, such as every 5 years [56]. Whether beneficiaries are offered a default, a simplified approach to selecting options, or access to trusted advisors, reducing the need to choose to every five years would allow beneficiaries to stick with a chosen plan for longer. Increasing the duration of Part D plans would stop insurance companies gradually making plans less valuable over time. Insurers are currently incentivized to ‘boil the frog’ and increase prices yearly to an extent which does not prompt mass switching but also soon puts beneficiaries in a position where there are better options on the market [57].

From January 2025, the Inflation Reduction Act will remove one behavioral factor impacting Part D beneficiaries’ health behavior and has the potential to significantly increase adherence [6]. Further policy tweaks informed by behavioral science may help beneficiaries to overcome barriers to switching Part D plans, thereby saving them money and reducing the risk of cost-related non-adherence. Of course, these policy tweaks need to be informed by evidence of what works, and what doesn’t work, for improving beneficiaries’ choices.

## Conclusions

This viewpoint highlighted several behavioral barriers that prevent Medicare Part D beneficiaries from switching to better plans, including information overload, low salience of benefits, and lack of trust or confidence. Addressing these barriers requires policies that simplify decision-making and offer trusted support to beneficiaries. Such changes are essential to help Medicare Part D achieve its goal of providing affordable and accessible prescription drug coverage, ultimately improving health outcomes and reducing costs for vulnerable populations. Across other domains of health policy, insights from behavioral science can provide insights into the interpretation of qualitative and quantitative data.

## Data Availability

Not applicable.
